# Liver Transplantation for Extra-Hepatic Manifestation of Hereditary Hemorrhagic Telangiectasia

**DOI:** 10.7759/cureus.27968

**Published:** 2022-08-13

**Authors:** Grace Park, Ashley E Stueck, Jordan Francheville, Joseph MacNeil, Julie H Zhu

**Affiliations:** 1 Department of Internal Medicine, Dalhousie University, Halifax, CAN; 2 Department of Pathology, Dalhousie University, Halifax, CAN; 3 Division of Digestive Care and Endoscopy, Dalhousie University, Halifax, CAN; 4 Faculty of Medicine, Royal College of Surgeons in Ireland, Dublin, IRL

**Keywords:** heart failure, transplant, osler-weber-rendu syndrome, hereditary hemorrhagic telangiectasia, primary biliary cholangitis

## Abstract

Hereditary hemorrhagic telangiectasia (HHT) is a rare genetic disorder distinguished by multiple arteriovenous malformations that can affect the liver and lungs, and additionally cause high-output heart failure. Effective medical treatment for HHT-related heart failure is limited. While most types of heart failure are contraindications in liver transplants, HHT-related high-output heart failure is an indication for a liver transplant. However, this is rarely performed as it poses a higher-than-average intraoperative risk. We present a case of a 57-year-old female patient with HHT and high-output heart failure from HHT who underwent a successful orthotopic liver transplant to significantly improve her heart function. Incidentally, the patient had a concomitant diagnosis of primary biliary cholangitis (PBC) from her explanted liver. We review the literature on liver transplants related to HHT and perioperative risks associated with heart failure and pulmonary hypertension that may be associated with both HHT and PBC.

## Introduction

Hereditary hemorrhagic telangiectasia (HHT), or Osler-Weber-Rendu syndrome, is an inherited disorder characterized by vascular malformations in liver, lung, brain, and gastrointestinal tract. Hepatic arteriovenous malformations (AVMs) lead to intrahepatic arteriovenous shunts, increased cardiac output, and heart failure [1]. Primary biliary cholangitis (PBC) is an autoimmune disease characterized by inflammation and progressive destruction of small intrahepatic bile ducts, resulting in fibrosis [2]. Each condition is rare and both occurring simultaneously has been described in only three case reports [3-5]. We describe the fourth such case, that of a 57-year-old woman with HHT and heart failure requiring a liver transplant. It is also the first described liver transplant in a patient with both HHT and PBC.

## Case presentation

A 57-year-old female was admitted to the cardiology ward with a 16-year history of progressive heart failure symptoms including orthopnea, dyspnea on exertion, and pedal edema. Her medical history included bleeding from the tongue and telangiectasias of the skin, tongue, and nasal caudal septa. She also had hypothyroidism, sicca syndrome, and chronic iron deficiency anemia from frequent epistaxis. She received weekly iron infusions for her anemia. Transthoracic echocardiogram revealed severe tricuspid regurgitation, dilated right atrium, hyperdynamic left ventricular ejection fraction with a normal right ventricular function, and elevated right ventricular systolic pressure of 42 mmHg (normal: 15-30 mmHg). Work-up included an MRI abdomen and CT chest that demonstrated multiple pulmonary and liver AVMs; therefore, HHT was suspected. Genetic testing revealed autosomal dominant activin receptor-like kinase 1 (ALK-1) mutation on chromosome 12q indicative of type 2 HHT. Her identical twin sister and her sister’s two daughters had similar symptoms and were subsequently diagnosed with HHT. Her heart failure symptoms were initially treated with two types of diuretics and metoprolol. However, she went on to have refractory symptoms, developed atrial fibrillation, and was unable to be treated with anticoagulation due to her epistaxis episodes.

She had normal liver biochemistry at the time of her diagnosis of HHT. Evidence of liver biochemical abnormality did not present until 10 years later when bloodwork revealed elevated alkaline phosphatase (ALP) at 200 IU/L (normal: 38-150 IU/L) with normal alanine transaminase and aspartate transaminase levels as well as a normal liver synthetic function panel. This was attributed to nitrofurantoin, which she had been exposed to the prior week. While aminotransferases and bilirubin normalized, ALP remained elevated at around 300-500 IU/L. Ensuing bloodwork revealed positive anti-nuclear antibody (ANA) titer and anti-mitochondrial antibody (AMA) titer at 1:160 with elevated IgM level. Magnetic resonance cholangiopancreatography (MRCP) showed a normal biliary tree and no evidence of splenomegaly or ascites. With these results and her history of sicca syndrome, she was suspected to have PBC, and was started on ursodeoxycholic acid (UDCA) for a clinical diagnosis of PBC. Unfortunately, she was intolerant of UDCA because of nausea, and was not considered for obeticholic acid at the time due to diagnostic uncertainty and a lack of a liver biopsy. Liver biopsy was not pursued due to the hepatic AVMs.

She continued to have complications from her HHT, including epistaxis, gastrointestinal AVMs, high-output heart failure, and an embolic stroke from atrial fibrillation. The heart failure was thought to be largely due to the extensive arteriovenous shunting in the liver. She was not a candidate for embolization due to significant hepatic AVM burden. Her heart failure symptoms such as orthopnea and marked limitation of activity secondary to dyspnea were refractory to diuretics and beta-blocker use. Fick calculated cardiac output was 7.25 L/min for a cardiac index of 4.56 L/min/m2. Her right-sided heart catheterization showed a normal mean pulmonary wedge pressure (PCW) of 10 mmHg and mean pulmonary arterial pressure of 19 mmHg.

**Figure 1:**
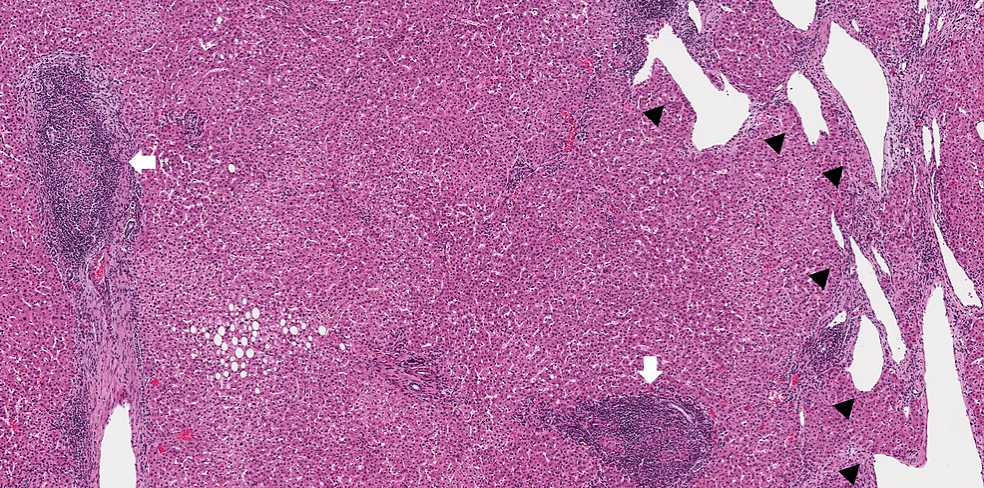
Explant liver pathology Abnormal and ectatic vascular structures, indicative of a shunt, are present (arrowheads). Portal tracts contain duct-centric inflammation and florid duct lesions (arrows). Hematoxylin and eosin (H&E), 5x.

Due to her high-output heart failure, cardiology and hepatology teams assessed the patient for a liver transplant and despite a low natural Model for End-stage Liver Disease (MELD) score, she was placed on the transplant list. She eventually received orthotopic liver transplantation. This was from a deceased donor and a duct-to-duct biliary anastomosis was performed. She was given methylprednisone, basiliximab, tacrolimus, and mycophenolate mofetil for immunosuppression induction. Perioperatively, she was closely followed by the cardiology team in the hospital to adjust her diuretic needs in the immediate post-transplant period. She required a reduced dose of furosemide post-transplant and was extubated on postoperative day two. There were no immediate post-transplant complications, and she was discharged home on postoperative day 13 with plans to follow up with cardiology and the liver transplant clinic. At her three-month follow-up, she was found to have hepatic artery thrombosis, but her liver synthetic function remained stable, and she was not retransplanted. A repeat echocardiogram showed improved tricuspid regurgitation (trace regurgitation) at two months post-transplant and significant clinical improvement in heart failure symptoms. Explant pathology demonstrated numerous intrahepatic vascular malformations and shunts with various degrees of associated congestive injury and fibrosis, up to cirrhosis (Figure 1). Due to shunting, there were frequent focal nodular hyperplasia (FNH) and FNH-like nodules. There was also duct-centric mixed portal inflammation with granulomatous destruction of small bile ducts, pathognomonic florid duct lesions, and <10% duct loss, consistent with PBC (Figure 1). She was started on low-dose UDCA to prevent PBC recurrence post-transplant.

## Discussion

HHT is an autosomal dominant disorder characterized by vascular malformations involving multiple organs. Presentations include epistaxis, gastrointestinal bleeding, and AVM-related complications [6]. Studies suggest prevalence from one in 5000 to 8000 [1].

There are two main types of HHT: HHT1 is characterized by mutations in the endoglin gene (chromosome 9), while HHT2 involves mutations in the ALK-1 (chromosome 12). Both involve the transforming growth factor beta (TGFB) signaling pathway [6]. Hepatic vascular malformations from HHT can be diffuse and vary from small telangiectasias to large AVMs. With time, the AVMs can increase in size and complexity [7]. Complications from arteriovenous shunting include high-output cardiac heart failure, pulmonary hypertension, portal hypertension, ischemic cholangiopathy, and hepatic encephalopathy [2]. HHT is diagnosed using the Curacao criteria; our patient met all four criteria (Table 1) [8]. There is little consensus on the treatment for HHT; however, liver transplantation is considered a radical but definitive treatment for medically-refractory liver or cardiac involvement. In this case, although she had an echocardiographic finding of mild to moderate pulmonary hypertension (42mmHg), right cardiac catheterization confirmed a normal pulmonary wedge pressure and arterial pressure, and was within the transplant parameter. The indication for liver transplant was primarily for refractory heart failure symptoms, driven by a high burden of AVMs in the liver [9].

**Table 1:** Curacao criteria for diagnosis of hereditary hemorrhagic telangiectasia HHT: Hereditary hemorrhagic telangiectasia, AVM: Arteriovenous malformation

Hereditary hemorrhagic telangiectasia criteria	
1. Epistaxis	Spontaneous, recurrent
2. Telangiectasias	Multiple, at characteristic sites: lips, oral cavity, fingers, nose
3. Visceral lesions	For example: gastrointestinal telangiectasia; pulmonary, hepatic, or cerebral AVMs
4. Family History	First-degree relative with HHT
Hereditary hemorrhagic telangiectasia diagnosis is:	
Definite	If ≥3 criteria are present
Possible or suspected	If 2 criteria are present
Unlikely	If 1 criterion is present

PBC is an autoimmune disease characterized by progressive inflammation and destruction of intrahepatic bile ducts, leading to fibrosis [2]. Prevalence is estimated at 39.2 per 100,000 persons, predominantly affecting females [10]. Characteristic findings of PBC are fatigue and pruritis. Diagnosis is outlined in Table 2 [11].

**Table 2:** Diagnosis of primary biliary cholangitis

Achieved when patients have at least two of the following three diagnostic criteria
1. Elevated alkaline phosphatase
2. Positive antimitochondrial antibodies, and/or
3. Evidence of nonsuppurative destructive cholangitis and destruction of interlobular bile ducts

Our patient had chronic ALP elevation, positive AMA titer, elevated IgM, and a history of sicca symptoms, making a clinical diagnosis of PBC possible. UDCA is a first-line therapy, however, intolerance or non-response to UDCA predicts disease progression and carries a poor prognosis [12]. Liver involvement from HHT can cause biliary tree ischemia [13] and ALP elevation [14]. However, it is difficult to confirm PBC and/or the co-existence of the two conditions without a liver biopsy.

Simultaneous PBC and HHT are rare, and this is the fourth described case. The concurrence is likely coincidental as there has been no reported genetic linkage between the two conditions. Currently, it is thought that exposure to infectious or inflammatory agents in genetically predisposed individuals initiates an immune reaction that causes PBC [2]. Known environmental risk factors for PBC include smoking and urinary tract infection (UTI), specifically with *Escherichia coli* [15]. Interestingly, our patient had both risk factors preceding her PBC diagnosis.

As previously mentioned, the pathogenesis of HHT is related to mutations that disrupt TGFB signaling [6]. Although heart failure is generally considered an important contraindication for a liver transplant, the case report highlights the exception to this rule when left to right shunts from hepatic AVMs are causes of high-output heart failure. The patient was considered a higher-risk transplant candidate due to her high-output heart failure and atrial fibrillation. However, there were no absolute contraindications and therefore she received a liver transplant to alleviate her heart failure symptoms. A literature review of liver transplantation for HHT shows that although it is increasing in frequency, HHT is still a rare indication for liver transplant. For example, in the United States, 39 HHT patients were reported to be listed and 24 were transplanted from 1998 to 2016 [16]. Recipients were predominantly female (84.6%), Caucasians (92.3%), had a low median MELD score (MELD=8), and half of the patients received a MELD exception score. The intraoperative mortality rate was 8.3%. The most common immediate post-transplant complications were hepatic artery thrombosis and hepatic vein thrombosis. Despite this, no patients required retransplant. Patient and graft survival rates at 48 months were 86% [16].

This case highlights a rare liver condition of HHT requiring a liver transplant to treat an extra-hepatic manifestation of HHT. The interdisciplinary approach is essential in optimizing perioperative risk and post-transplant survival. It reports the first successful liver transplant in such a patient with HHT and an incidental finding of PBC.

## Conclusions

This case highlights a rare liver condition of HHT requiring a liver transplant to treat an extra-hepatic manifestation of HHT. It also reports the first successful liver transplant in such a patient with HHT and an incidental finding of PBC. Cases of HHT and PBC occurring simultaneously have been reported extremely rarely. This is likely coincidental as there has been no reported genetic linkage between the two conditions. The interdisciplinary approach is essential in optimizing perioperative risk and post-transplant survival.
